# Supplementary material for: Association between Feelings of Trust and Security with Subjective Health among Mexican Migrants in the New York City Area
Source: Int J Environ Res Public Health. 2023 Feb 8;20(4):2981. doi: 10.3390/ijerph20042981 (PMC9966475; doi:10.3390/ijerph20042981)
Supplement: Supplementary file 1 [file ijerph-20-02981-s001.zip › ijerph-2002203-Supplementary.pdf]

Supplementary Material

Table S1. Logistic regression on Self-reported Health Status (5 model variations).

	**(1) Separate items**	**(2) Security item**	**(3) Trust score**	**(4) Trust score and neighborhood safety**	**(5) Trust score * Neighborhood safety**	**(6) Trust and safety score**
Age (years)	1.024 [0.994, 1.056]	1.011 [0.983, 1.041]	1.012 [0.984, 1.042]	1.019 [0.989, 1.050]	1.018 [0.989, 1.050]	1.016 [0.987, 1.047]
Sex	1.334 [0.794, 2.249]	1.392 [0.850, 2.287]	1.292 [0.782, 2.140]	1.305 [0.784, 2.179]	1.296 [0.778, 2.165]	1.318 [0.794, 2.195]
Household composition	0.755 [0.445, 1.276]	0.727 [0.440, 1.195]	0.797 [0.478, 1.324]	0.741 [0.438, 1.245]	0.731 [0.432, 1.231]	0.762 [0.454, 1.275]
Employment status	1.407 [0.746, 2.675]	1.514 [0.845, 2.739]	1.427 [0.779, 2.638]	1.334 [0.716, 2.507]	1.330 [0.711, 2.503]	1.431 [0.777, 2.659]
Length of residence in the US (years)	0.976 [0.938, 1.014]	0.989 [0.954, 1.026]	0.991 [0.955, 1.028]	0.985 [0.949, 1.024]	0.985 [0.948, 1.023]	0.985 [0.949, 1.023]
Diagnosed comorbidities	2.782** [1.655, 4.754]	2.522** [1.544, 4.172]	2.767** [1.671, 4.656]	2.687** [1.609, 4.559]	2.705** [1.618, 4.593]	2.704** [1.626, 4.565]
Has valid health insurance in the US	1.117 [0.675, 1.847]	1.142 [0.712, 1.833]	0.962 [0.591, 1.562]	1.033 [0.629, 1.692]	1.030 [0.627, 1.688]	0.996 [0.609, 1.625]

How frequently has gotten medical care when needed	1.644 [0.986, 2.770]	1.384 [0.853, 2.259]	1.711+ [1.044, 2.835]	1.605 [0.969, 2.685]	1.646 [0.989, 2.768]	1.701+ [1.034, 2.829]
**Subjective measures of trust and safety**						
I can trust most people in my community	0.533+ [0.301, 0.929]	.	.	.	.	.
I can get help from my neighbors whenever I need it	2.108* [1.154, 3.924]	.	.	.	.	.
I feel safe when I walk alone at night in my community	1.144 [0.511, 2.569]	.	.	.	.	.
Rating of neighborhood safety	3.520* [1.393, 9.734]	3.586** [1.719, 8.091]	.	3.383* [1.450, 8.621]	5.157* [1.396, 22.649]	.

Three-item measure of trust	.	.	1.177 [0.924, 1.505]	1.028 [0.783, 1.348]	1.350 [0.656, 2.763]	.
Three-item measure of trust * Rating of neighborhood safety	.	.	.	.	0.729 [0.338, 1.575]	.
Four-item composite measure of trust and safety	.	.	.	.	.	1.257+ [1.021, 1.557]
Num.Obs.	225	240	227	225	225	225
AIC	306.7	325.9	316.1	308.6	310.1	310.6
BIC	351.1	360.7	350.3	346.1	351.1	344.7
Log.Lik.	-140.331	-152.939	-148.03	-143.284	-143.048	-145.286
RMSE	0.47	0.47	0.48	0.47	0.47	0.48

OR calculated at 90% confidence level. Exponentiated coefficients; 95% confidence intervals in brackets. + $p < 0.1$, * $p < 0.05$, ** $p < 0.01$

Table S2. Regression process on Self-reported Health Status by each of the four items on trust and safety.

Dependent: Self-Reported Health Status	Univariable Regressions (each Coefficient alone)	Multivariable Regression	Multivariable Regression with Coefficients which had a p-Value < 0.2
Age (years)	1.00 (0.97-1.03, p=.961)	1.02 (0.99-1.06, p=.195)	
Sex	1.25 (0.74-2.12, p=.403)	1.33 (0.72-2.48, p=.362)	
Household composition	0.74 (0.42-1.29, p=.283)	0.76 (0.40-1.41, p=.380)	
Employment status	1.29 (0.69-2.41, p=.429)	1.41 (0.66-3.00, p=.378)	
Length of residence in the US (years)	0.99 (0.96-1.02, p=.610)	0.98 (0.93-1.02, p=.296)	
Diagnosed comorbidities	2.42 (1.41-4.16, p=.001)	2.78 (1.49-5.21, p=.001)	2.81 (1.55-5.08, p<.001)
Has valid health insurance in the US	1.17 (0.68-1.99, p=.572)	1.12 (0.61-2.03, p=.717)	
How frequently has gotten medical care when needed	1.32 (0.78-2.25, p=.296)	1.64 (0.89-3.04, p=.112)	1.70 (0.94-3.06, p=.078)
I can trust most people in my community	0.99 (0.58-1.70, p=.985)	0.53 (0.27-1.04, p=.066)	0.59 (0.32-1.12, p=.106)
I can get help from my neighbors whenever I need it	1.83 (0.99-3.38, p=.055)	2.11 (1.02-4.36, p=.044)	2.03 (1.01-4.10, p=.047)
I feel safe when I walk alone at night in my community	1.93 (0.95-3.91, p=.069)	1.14 (0.44-2.98, p=.783)	
Rating of neighborhood safety	3.99 (1.56-10.23, p=.004)	3.52 (1.12-11.11, p=.032)	3.68 (1.35-10.00, p=.011)

OR calculated at 95% confidence level.
